# AGE AND GENDER IMPACTS ENDORSEMENT OF NOSTALGIA ACROSS SEVEN YEARS

**DOI:** 10.1093/geroni/igae098.2700

**Published:** 2024-12-31

**Authors:** Jennifer Turner, Tim Wildschut, Constantine Sedikides

**Affiliations:** University of Hawaii Hilo, Hilo, Hawaii, United States; University of Southampton, Southampton, England, United Kingdom; University of Southampton, Southampton, England, United Kingdom

## Abstract

The experience of nostalgia (a bittersweet longing for one’s past) is common amongst people of all ages (Hepper et al., 2021; Juhl et al., 2020). Recent work described age-group differences in the frequency of nostalgia, such that older adults reported more nostalgia compared to middle-aged and younger adults (Turner & Stanley, 2021). Some have posited that age-related differences in nostalgia are founded on changing emotion-regulation goals and future time perspective in later life (Hepper et al., 2021), yet one problem has been the emphasis on cross-sectional studies which limit understanding of potential developmental change in nostalgia. This study extends previous work by examining the relevance of individual differences (i.e., age and gender) for endorsement of nostalgia in a nationally-representative sample of Dutch-speaking participants (N=2,664, 53.5% women) using a longitudinal design from the LISS panel (8-waves collected 2012-2019). At baseline, participants’ ages ranged between 9-93 years (M=48.59, SD=18.07). At each wave, participants completed the 7-item Southampton Nostalgia Scale (Sedikides et al., 2015), which assesses how frequently, and how much, one values the experience of nostalgia (Wang et al., 2023). As expected, analyses supported both between-person age differences, with older adults endorsing greater nostalgia than younger ones, and within-person age-related increases in nostalgia. Gender differences also emerged, such that women were more nostalgic than men on average, but this was qualified by a significant interaction with age: men (but not women) demonstrated increases in nostalgia with age. Results are interpreted through the lens of Socioemotional Selectivity Theory (Carstensen, 2021).

